# ROCIP: robust continuous inertial position tracking for complex actions emerging from the interaction of human actors and environment

**DOI:** 10.1007/s10489-025-06409-1

**Published:** 2025-03-01

**Authors:** Xinyu Hou, Jeroen Bergmann

**Affiliations:** Department of Engineering Science, University of Oxford, Parks Road, Oxford, OX1 3PJ UK

**Keywords:** Machine learning, Inertial navigation, Pedestrian dead reckoning, Deep neural network, Inertial measurement unit, Wearable sensors

## Abstract

Inertial navigation is advancing rapidly due to improvements in sensor technology and tracking algorithms, with consumer-grade inertial measurement units (IMUs) becoming increasingly compact and affordable. Despite progress in pedestrian dead reckoning (PDR), IMU-based positional tracking still faces significant noise and bias issues. While traditional model-based methods and recent machine learning approaches have been employed to reduce signal drift, error accumulation remains a barrier to long-term system performance. Inertial tracking’s self-contained nature offers broad applicability but limits integration with a global reference frame. To solve this problem, a system that could “introspect its error” and “learn from the past” is proposed. It consists of a neural statistical motion model that regresses both poses and uncertainties with DenseNet, which are then fed into Rao-Blackwellised particle filter (RBPF) for calibration with a probabilistic transition map. An inertial tracking dataset with head-mounted IMUs was collected, including walking and running with different speeds while allowing participants to rotate their heads in a self-selected manner. The dataset consisted of 19 volunteers that generated 151 sequences in 4 scenarios with a total time of 929.8 min. It was shown that our proposed method (ROCIP) outperformed the leading methods in the field, with a relative trajectory error (RTE) of 4.94m and absolute trajectory error (ATE) of 4.36m. ROCIP could also solve the problem of error accumulation in dead reckoning and maintain a small and consistent error during long-term tracking.

## Introduction

In recent years, localization technologies have advanced rapidly across various domains, including autonomous driving, unmanned aerial vehicles (UAVs), and domestic robots like vacuum cleaners. Human positional tracking has also benefited from these advancements, finding applications in navigation [1], healthcare [2], tracking [3], smart homes, sports, and emergency services [4]. Common techniques include GNSS, cameras, WiFi, cellular networks, Bluetooth, RFID, Ultra-wideband, infrared, and radar. While these methods offer high accuracy, their reliance on external signals or infrastructures limits their use in complex environments. Moreover, carrying heavy equipment for extended periods in dynamic environments is impractical for pedestrians. In such cases, Inertial Navigation Systems (INS) using low-cost, low-power Inertial Measurement Units (IMUs) are advantageous as they are self-contained and operate without external infrastructure. IMUs are particularly useful in environments with limited or no external signals, such as underground, in the wilderness, or during emergency events. However, their sensitivity to variations in human behavior, such as gait diversity, can reduce accuracy.

Despite these advantages, consumer-grade MEMS IMUs face significant limitations. Their low precision and the inherent errors from double integration in Strap-down Inertial Navigation Systems (SINS) make them prone to rapid error accumulation. Numerous methods have been proposed to address these issues in low-cost IMU-based positioning.

Traditional human inertial tracking relies on model-based pedestrian dead reckoning (PDR), which divides trajectory estimation into step detection, step length, and heading estimation [5]. However, these models use simplified equations and specific parameters, leading to limited robustness in diverse environments and among different users.

Recent advancements in computational resources have enabled machine learning to tackle complex, high-dimensional problems, showing promise in fields such as natural language processing [6], image processing [7], medicine [8], electricity [9], and healthcare [10]. This capability extends to human inertial tracking. Early studies [11–15] used machine learning for IMU-based human odometry and velocity vector regression to improve trajectory estimation. Advanced neural networks, such as ResNet, LSTM, and Temporal Convolutional Networks, have also been employed to estimate human movement.

**Fig. 1:**
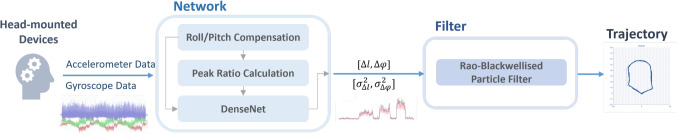
Overview of the system. Raw accelerometer and gyroscope data form the input from the head-mounted IMUs. The position and uncertainties are estimated by DenseNet and form the input for the particle filter

Sensor placement is a critical factor in inertial navigation. Different placements produce varying IMU signal patterns, affecting model performance. A survey [16] revealed that users prefer small, discreet sensors embedded in everyday objects, with head-mounted systems offering considerable potential due to their integration with items like glasses, earphones, or helmets. However, head-mounted sensors remain underutilized, with most studies placing sensors on the lower limbs or in mobile phones [17], despite the need for alternatives in activities where carrying a phone is impractical.

Previous studies have addressed challenges in inertial tracking with head-mounted sensors, such as positional estimation during head motion and error accumulation over time [5, 18, 19]. However, new questions have emerged with the use of machine learning techniques, particularly regarding prediction uncertainty. Assessing uncertainty is crucial for understanding how much trust to place in the results, especially in safety-critical scenarios where inaccurate positional data could pose risks. Deterministic results with unknown errors may lead to poor decision-making, while quantified uncertainty enables better risk assessment and integration with decision models or other data sources (e.g., GPS, cameras). Moreover, uncertainty estimation can improve the application of Kalman or particle filters. Previous research on uncertainty in deep learning [20, 21] highlights its potential value for localization problems.

Understanding uncertainty in estimates is essential, but addressing the complexity of human mobility is equally important for real-world applications. Many pedestrian inertial navigation methods lack long-term robustness in varied, real-world environments, despite claims of high accuracy in controlled studies. A major limitation is the unpredictability of human behavior, including transitions between locomotion modes (e.g., walking, jogging, running) and irregular motions such as side-stepping, stopping, or starting unexpectedly [22]. Most traditional methods, like PDR, fail in real-world scenarios as they model only basic gait patterns observed under controlled conditions. Machine learning, however, offers the potential to capture these complex, realistic motions autonomously. Some studies have begun addressing this gap by incorporating diverse motion types, including walking, running, side-stepping, and stair climbing [14, 23, 24].

Nonetheless, error accumulation remains an inevitable problem in dead reckoning if there is no opportunity for calibration with the true positions in the real world. To overcome this complication, common calibration approaches have been used, which include, for example, the application of a camera or RFID. It is obvious that these solutions introduce external infrastructures and/or extra devices. It provides a solution that is no longer self-contained and reduces the ability to widely utilize this kind of technology. As for using IMUs only, ongoing calibrations for long-term monitoring with the real world is impossible. However, there exists a way to eliminate the error accumulation by calibrating the system with its own prior trajectories. Similar ideas have been used in [25, 26]. However, these studies were conducted only in situations where volunteers were walking at a constant pace. Furthermore, fixed uncertainty parameters needed to be manually set, which reduces their robustness and generalizability across various users, motions, and environments.

In this study, ROCIP, an inertial navigation system for a head-mounted IMU, is proposed. It uses a probabilistic DenseNet model to estimate both pose and uncertainty in a mixed supervised and unsupervised way and is tightly coupled with an Rao-Blackwellised particle filter (RBPF) for optimal estimation. The system is tested for “long-term” tracking with various head motions and different speeds of walking and running. The main contributions are as follows.

- We propose a network specially designed for inertial navigation with head-mounted IMUs, based on special input features and outputs that include both displacement and uncertainty, which is trained in a mix of supervised and unsupervised way.
- We propose a complete state estimation system combining the neural network with the RBPF to control the error accumulations in long-term localization.
- We introduce an inertial tracking dataset with head-mounted IMUs, with a total length of 929.8min, 151 sequences, and 19 subjects, which includes human walking and running with different speeds and random head rotations.

ROCIP was compared with two other well-defined methods for head-mounted sensors, and the absolute and relative errors were computed across all three approaches.

## Methods

The system consists of two main components: (i) network and (ii) filter. Figure 1 shows the main structure of the system.

### Preprocessing

The raw data from the IMU sensor were first transformed into a normalized coordinate system in which the *z* axis is aligned with the gravity direction, while there is no gravity component on the normalized *x* axis and the *y* axis. Equation (1,2)-(4) show the normalizing process where $a_{raw}, \omega _{raw}$ are the raw accelerometer and gyro data, and $a_{norm}, \omega _{norm}$ are the normalized acceleration and angular velocity data.

1 $$\begin{aligned}  &   a_{norm} \!=\! R_{a}^{-1} \cdot a_{raw} \end{aligned}$$

2 $$\begin{aligned}  &   R_{a} \!=\! R_{x}(\phi )R_{y}(\theta ) = \begin{bmatrix} \cos \theta &  0 &  -\sin \theta \\ \sin \phi \sin \theta &  \cos \phi &  \sin \phi \cos \theta \\ \cos \phi \sin \theta &  -\sin \phi &  \cos \phi \cos \theta \end{bmatrix} \end{aligned}$$

3 $$\begin{aligned} \omega _{norm} = R_{\omega }^{-1} \cdot \omega _{raw} \end{aligned}$$

4 $$\begin{aligned} R_{\omega } = \begin{bmatrix} 1 &  0 &  -\sin \theta \\ 0 &  \cos \phi &  \sin \phi \cos \theta \\ 0 &  -\sin \phi &  \cos \phi \cos \theta \end{bmatrix} \end{aligned}$$

Then the peak ratio features were calculated, which were specifically designed for tracking with head-mounted devices to distinguish head rotation from whole-body rotation [18]. Two primary body movements generate regular acceleration waves during walking: (i) side-to-side body swinging and (ii) front-to-back linear acceleration from stepping, which are in perpendicular directions and with different frequency. When the head is held straight, only stepping signal is recorded on the IMU’s forward axis, while both stepping and side swing signal appear when the head is turned sideways. This distinction helps identify whether a rotation results from head movement without a change in walking direction or from a change in walking direction with head-body alignment. Equation (5) shows the calculation of peak ratio $P_{ratio}$, where $P_{swing}, P_{stepping}$ are the peak magnitudes in the frequency domain generated by swing and stepping.

5 $$\begin{aligned} P_{ratio} = \frac{P_{swing}}{P_{stepping}} \end{aligned}$$

The peak ratios and normalized IMU data form the input into the subsequent neural network.

### DenseNet

**Fig. 2:**
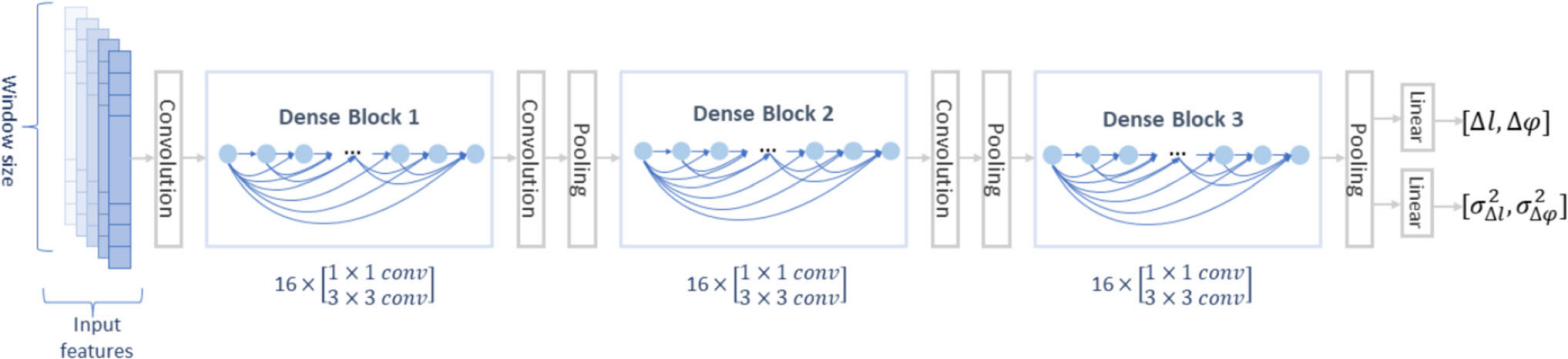
Architecture of DenseNet-BC 100 that is used in the study

Dense Convolutional Network (DenseNet) [27] is an improved version of Convolutional Neural Networks (CNNs). CNNs have been the dominant machine learning approach in image processing tasks for over 10 years. More recently, improvements in computer hardware have enabled the training of truly deep CNNs. Yet, the problem of gradient vanishing emerges as the layers are getting deeper. ResNet [28] was proposed to solve this problem by passing signals from one layer to the next via identity connections, and it has been used in RoNIN [13] for inertial navigation. DenseNet connects all layers directly with each other to ensure maximum information flow between layers in the network. It was evaluated on four highly competitive object recognition benchmark tasks and obtained significant improvements over the state-of-the-art with less computational effort. In this research, a DenseNet-BC 100 was adopted as the backbone, which is a DenseNet with bottleneck layers and compression, with 100 convolutional layers. It has three dense blocks. Each block has 16 layers, with each layer having a bottle neck layer and a convolutional layer. DenseNet has been adapted to fit this research, with the layers reshaped to process 1D information. The input and output have also been redesigned for this research. The input could be recognised as a 1D image with a shape of $window\_size \times 1$ and the channels are input features, which could be represented as $(a_{norm}, \omega _{norm}, P_{ratios})_{8*120}$. It outputs the moving distance and orientation variation $(\Delta l, \Delta \varphi )_{2}$, with the uncertainty of both in a time window. Figure 2 shows the architecture of the adapted neural network.

### Uncertainty estimation

In DenseNet, the model outputs are the estimated polar vectors and variances:

6 $$\begin{aligned} [\hat{\varvec{y}}, \hat{\varvec{\Sigma }}] = f_{\theta }(\varvec{x}) \end{aligned}$$

where $$\hat{\varvec{y}} = [\Delta \hat{l}, \Delta \hat{\varphi }]$$ and $$\hat{\varvec{\Sigma }} = diag(\sigma _{\Delta l}^{2}, \sigma _{\Delta \varphi }^{2})$$.

Assuming that $\varvec{y}$ follows a multivariate normal distribution:

7 $$\begin{aligned} \varvec{y} \sim \mathcal {N}_{2}(\hat{\varvec{y}}, \hat{\varvec{\Sigma }}) \end{aligned}$$

with a probability density function:

8 $$\begin{aligned} p(\varvec{y}|f_{\theta }(\varvec{x})) = \frac{1}{\sqrt{(2\pi )^{2}|\hat{\varvec{\Sigma }}|}} \exp {(-\frac{1}{2} (\varvec{y} - \hat{\varvec{y}})^{T} \hat{\varvec{\Sigma }}^{-1}(\varvec{y} - \hat{\varvec{y}}))} \end{aligned}$$

The optimal neural network weights $\theta ^{*}$ could be approximated by log-likelihood:

9 $$\begin{aligned} {\begin{matrix} \theta ^{*} & = \arg \max _\theta p(\varvec{y}|f_{\theta }(\varvec{x})) \\ & = \arg \min _\theta -2\log p(\varvec{y}|f_{\theta }(\varvec{x})) \\ & = \arg \min _\theta \log {|\hat{\varvec{\Sigma }}|} + (\varvec{y} - \hat{\varvec{y}})^{T} \hat{\varvec{\Sigma }}^{-1}(\varvec{y} - \hat{\varvec{y}}) \end{matrix}} \end{aligned}$$

To make regression more stable, $s_l$ and $s_\varphi$ are predicted by the neural network instead of $\sigma _{\Delta l}^{2}, \sigma _{\Delta \varphi }^{2}$, where $s_l = \log {\sigma _{\Delta l}^{2}}$ and $s_\varphi = \log {\sigma _{\Delta \varphi }^{2}}$ [21]. Thus, the loss function of neural network could be defined as:

10 $$\begin{aligned} \begin{aligned} \mathcal {L}&= \text {log}{|\hat{\varvec{\Sigma }}|} + (\varvec{y} - \hat{\varvec{y}})^{T} \hat{\varvec{\Sigma }}^{-1}(\varvec{y} - \hat{\varvec{y}})\\&= \log {\sigma _{\Delta l}^{2}} + \log {\sigma _{\Delta \varphi }^{2}} + \frac{(\Delta l - \Delta {\hat{l}})^2}{\sigma _{\Delta l}^{2}} + \frac{(\Delta \varphi -\Delta \hat{\varphi })^2}{\sigma _{\Delta \varphi }^{2}}\\&= s_l + s_\varphi + \frac{(\Delta l - \Delta {\hat{l}})^2}{\exp {s_l}} + \frac{(\Delta \varphi -\Delta \hat{\varphi })^2}{\exp {s_\varphi }} \end{aligned} \end{aligned}$$

The loss function shows that the network training contains a mixture of supervised and unsupervised parts. The estimated displacement and rotation $\Delta \hat{l}, \Delta \hat{\varphi }$ have their corresponding ground truth $\Delta l, \Delta \varphi$, so they could be optimised in a supervised way. While there is no ground truth for estimated uncertainty, the estimation of uncertainty is optimised in an unsupervised way.

### Rao-Blackwellised particle filter

Path inference uses the Rao-Blackwell particle filter of the FastSLAM algorithm [29], which decomposes the SLAM problem into a pedestrian localisation problem and a mapping problem conditioned on the estimated poses. Based on the conditional independence property of the SLAM, the pose posterior could be factorized as:

11 $$\begin{aligned} p(\textbf{P}_{0:k},\textbf{M}|\textbf{Z}_{1:k}) = p(\textbf{M}|\textbf{P}_{0:k}) \cdot p(\textbf{P}_{0:k}|\textbf{Z}_{1:k}) \end{aligned}$$

where **P** and **M** represent the pose and the map estimated in the current Bayes inference step. And ${\textbf {Z}}_{k}$, which is intrinsically equal to the $\hat{\varvec{y}}$ estimated from the previous neural network, now recognised as a noisy measurement of the difference between ${\textbf {P}}_{k-1}$ and ${\textbf {P}}_{k}$. The pose in time step *k* could be estimated recursively from the last with the first pose $\textbf{P}_0$ set arbitrarily:

12 $$\begin{aligned} p(\textbf{P}_{0:k}|\textbf{Z}_{1:k}) \propto p(\textbf{Z}_k|\textbf{P}_{k-1:k}) \cdot p(\textbf{P}_{k}|\textbf{P}_{0:k-1}) \cdot p(\textbf{P}_{0:k-1}|\textbf{Z}_{1:k-1}) \end{aligned}$$

The first factor in (12), $p(\textbf{Z}_k|\textbf{P}_{k-1:k})$, is the likelihood function of the pose difference estimation in the current part, which is expected to approximate to the real value $\varvec{y}$. Therefore it is proportional to $p(\varvec{y}|\hat{\varvec{y}})$, which is the posterior and also proportional to Function (8). This likelihood factor is used to sampling the particles in each step from a Gaussian distribution $\mathcal {N}_{2}(\varvec{\mu }_k, \varvec{\Sigma }_k)$, where

$$\begin{aligned} \varvec{\mu }_k&= \hat{\varvec{y}}_k = [\Delta l_k, \Delta \varphi _k]\\ \varvec{\Sigma }_k&= diag(\sum _{i=0}^{k} \sigma _{\Delta l_i}^2,\sum _{i=0}^{k} \sigma _{\Delta \varphi _i}^2) \end{aligned}$$

The second factor in (12), $p(\textbf{P}_{k}|\textbf{P}_{0:k-1})$, is the pose transition probability, which is used in the particle weight update (13). In this section, the two-dimensional space was divided into a grid of adjacent hexagons of a given radius. Each hexagon $\tilde{h}$ has six edges. Each time a particle makes a transition across an edge $\tilde{e}$ will be counted up and recorded in the number of transition times $N_{\tilde{h}}^{\tilde{e}}$. For each hexagon, the number of transition times of six edges is recorded in a local map $\textbf{M}_{\tilde{h}}$, which is a vector of length 6. All $\textbf{M}_{\tilde{h}}$ of every hexagon comprise the probabilistic transition map $\textbf{M}$. When updating the map after each step, if an edge was crossed, the transition times of this edge will increase in both the incoming and outgoing hexagons. The transition times recorded in the map are then used in the particle weight update:

13 $$\begin{aligned} w_k^i \propto p(\textbf{P}_{k}|\textbf{P}_{0:k-1}) \cdot w_{k-1}^i \propto \frac{N_{\tilde{h}}^{\tilde{e}} + {\alpha }_{\tilde{h}}^{\tilde{e}}}{N_{\tilde{h}} + {\alpha }_{\tilde{h}}} \cdot w_{k-1}^i \end{aligned}$$

where $w_k^i$ denotes the weight of the *i*-th particle at step *k*, $N_{\tilde{h}}$ is the sum of the crossed times of all 6 edges of the outgoing hexagon $\tilde{h}$ in *i*-th particle’s map counters. ${\alpha }_{\tilde{h}}^{\tilde{e}}$ and ${\alpha }_{\tilde{h}} = \sum _{e=0}^{5}{\alpha }_{\tilde{h}}^{\tilde{e}}$ are the prior counts.

With the above functions, the sequential Bayesian estimation will be conducted in a loop of particle sampling, weight updating, resampling, and probabilistic transition map updating. The RBPF has significant advantages over traditional particle filters due to its ability to combine particle filtering with analytical marginalization, which improves computational efficiency and accuracy in certain scenarios.

## Data collection

**Fig. 3:**
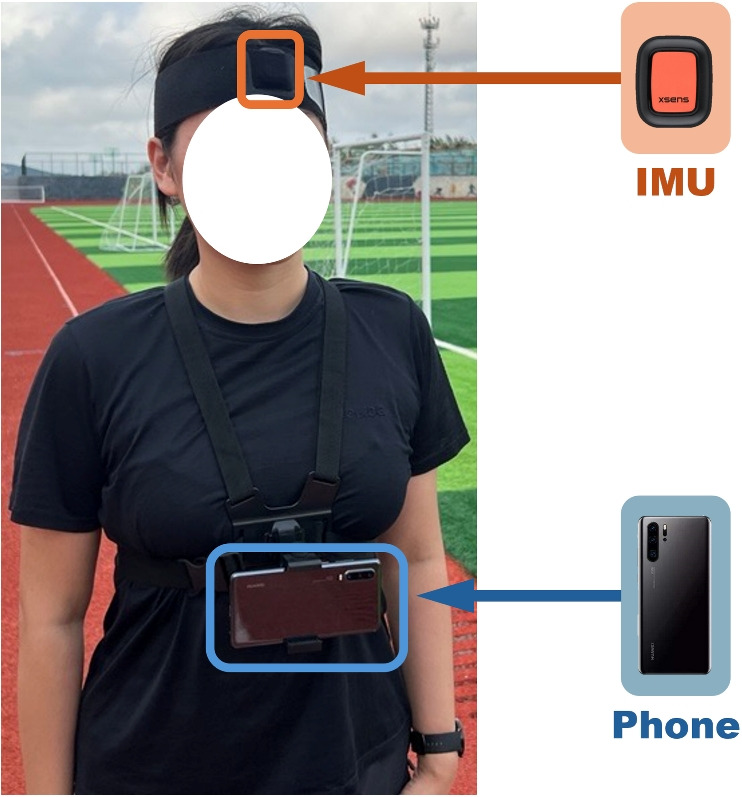
Experimental setup for data collection: A participant equipped with an Xsens IMU sensor mounted on the forehead using a headband (highlighted in orange) and a smartphone secured on the chest harness for synchronized data recording (highlighted in blue)

Xsens Dot sensors (Xsens Technologies BV, Enschede, Netherlands) were used in the experiments with a recording frequency of 60Hz. An Xsens Dot was firmly attached to the forehead using a strap that was adjusted for each subject. A phone was also firmly attached on the chest with straps to provide reference data. The device setup is shown in Fig. 3. An application developed in Unity 3D game engine (Unity Technologies, San Francisco, CA, USA) based on Google ARCore was installed in the phone for ground truth collection.

The data collection was conducted in four different environments with different path sizes and trajectories to ensure more varied patterns were captured. This should help to clarify the eternal validity of the results obtained. The trajectories included straight routes, curves, and turnings with different angles, which increased the complexity and diversity of the tests. There were 19 volunteers participating in the experiments with 11 males and 8 females. The participant’s ages ranged from 22 to 50 years. Ethical approval was obtained from the University Ethics Committee. Subjects were asked to walk and run with different speeds on the predetermined trajectories, whilst rotating their head in a random manner.

A total of 151 sequences of data were collected, with a total duration of 929.8 minutes. Each dataset contained both the IMU data from the Xsens Dots, as well as the ground truth trajectory and orientation data from the phone.

## Results

The proposed methods was trained on the University of Oxford Advanced Research Computing (ARC) facility [30], with the dataset split into a training, test and validation set (70%, 15%, 15%). During training, it adopted the stochastic gradient descent (SGD) optimizer ($weight\_decay = 0.0001, momentum = 0.9$) and multi-Step Learning Rate (MultiStepLR) scheduler ($milestones = [50, 75], gamma=0.1$), with a initial learning rate of 0.001, batch size of 128, and 100 epoches. Two published methods for tracking with a head-mounted IMU were chosen for comparison: a PDR method [5] using peak detection to detect steps, with complementary filter to determine orientations and Weinberg model to detect step lengths; and HINNet [18], which uses Bi-LSTM and peak ratio features. Quantitative analysis utilized three metrics shown in Table 1.

**Table 1:** Quantitative analysis metrics in this study

Metrics	Equations	Descriptions
ATE (*m*)	$\text {ATE}_{pos} =(\frac{1}{N}\sum _{i=0}^{N-1}\Vert \Delta \textbf{p}_{i}\Vert ^{2})^{\frac{1}{2}}$	Absolute trajectory error. ATE is the root mean square error (RMSE) between the whole ground truth trajectory and the estimated trajectory.
RTE (*m*/$\Delta t$)	$\text {RTE}_{pos} = (\frac{1}{N}\sum _{i=0}^{N-1}\Vert \textbf{p}_{e}- \Delta \textbf{p}_0 - \text {R}_{e}(\hat{\text {R}}_{e}^{\prime })^{\textsf{T}}\hat{\textbf{p}}_{e}^{\prime }\Vert ^{2})^{\frac{1}{2}}$	Relative trajectory error. RTE is defined as the average RMSE over a fixed time interval (1 minute in this study) with alignments of the initial states.
Distance error rate ($\%$)	$\text {Error}_l = |\text {Distance}_{real} - \text {Distance}_{est}| / \text {Distance}_{real}$	Drift of the estimated total distance.

It should be noted that RTE and ATE are standard position evaluation metrics in navigation [31]. RTE, ATE and percentage error of total distance covered for each method are summarised in Table 2.

**Table 2:** Relative trajectory error (RTE), absolute trajectory error (ATE), and percentage error of total distances of proposed and comparing methods

Methods	RTE(*m*)	ATE(*m*)	Distance Error (%)
PDR	7.99	13.85	7.26
HINNet	5.46	13.41	0.46
ROCIP	**4.94**	**4.36**	**0.23**

**Best** results are highlighted

**Fig. 4:**
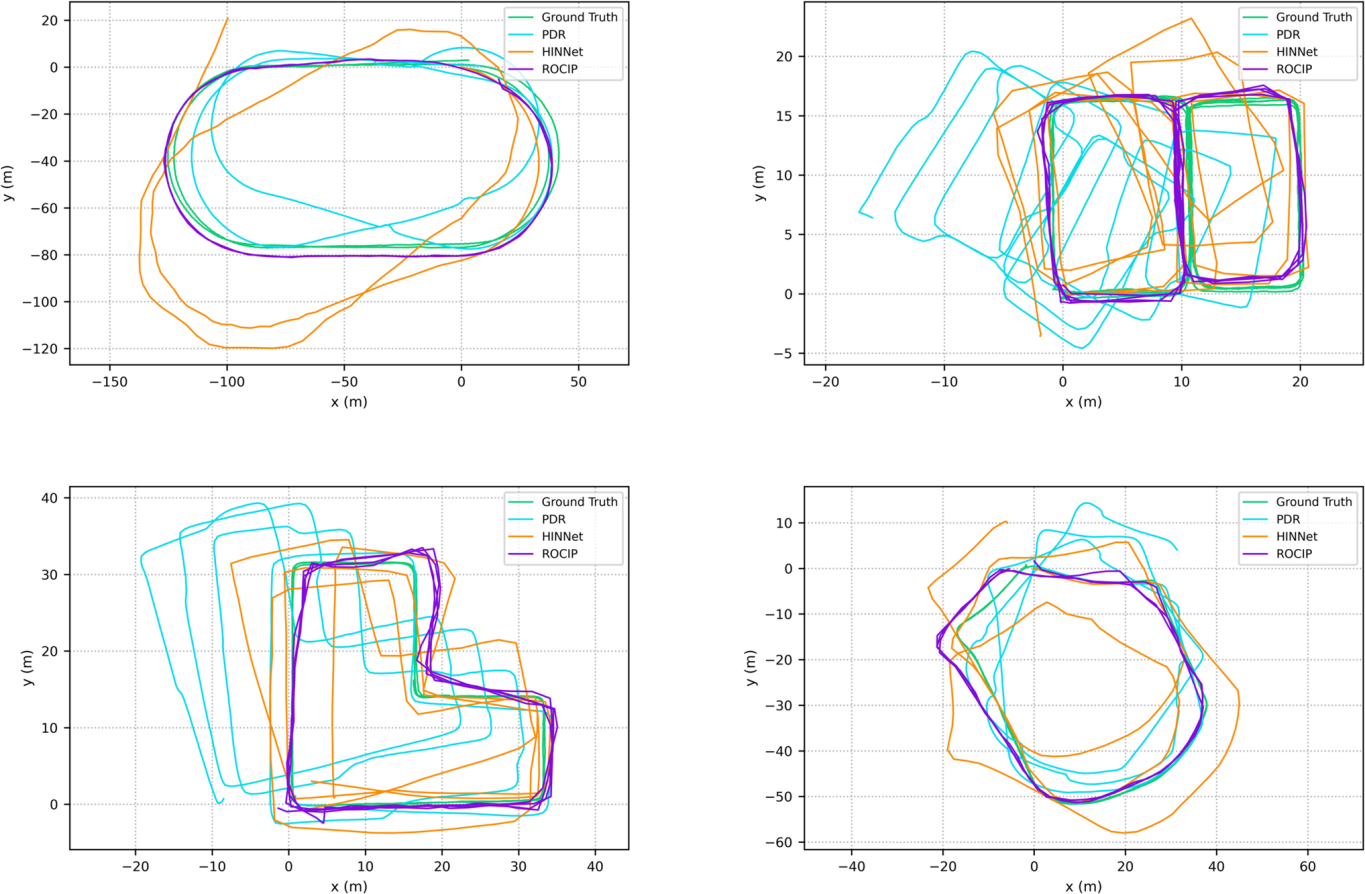
Estimated trajectories of different methods on four different paths. The green lines represent the ground truths. Orange lines show trajectories estimated by HINNet. Blue lines are the trajectories generated by the PDR. Purple lines are proposed ROCIP trajectories

The estimated trajectories of different methods in the 4 scenarios are shown in Fig. 4.

**Fig. 5:**
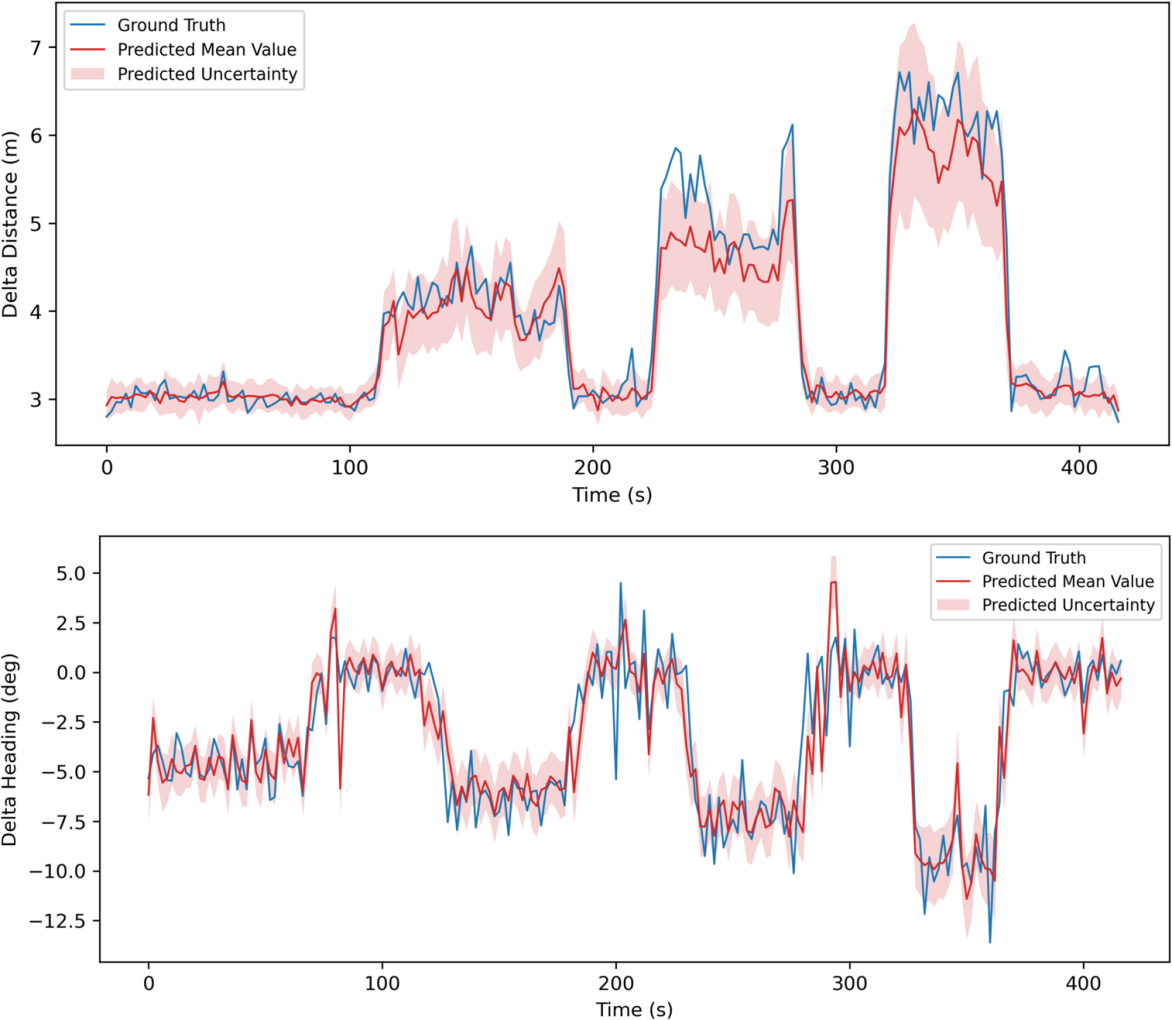
Estimated delta distance and heading in each 2 *s* interval from probabilistic DenseNet – **walking and running at various speeds**. Blue lines represent the Ground Truth. Red lines are mean values estimated by DenseNet. Light red zones show the estimated uncertainty $\sigma$

**Fig. 6:**
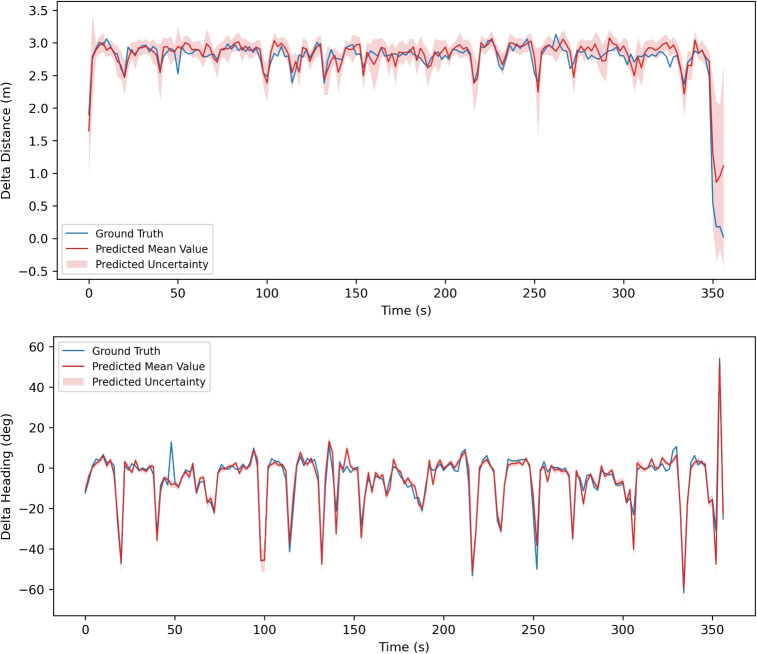
Estimated delta distance and heading in each 2 *s* interval from probabilistic DenseNet – **walking with constant pace**. Blue lines represent the Ground Truth. Red lines are mean values estimated by DenseNet. Light red zones show the estimated uncertainty $\sigma$

Figures 5 and 6 show the output of probabilistic neural network. The motion represented in Fig. 5 is walking and running in various speed. While Fig. 6 shows the motion with only walking with constant pace.

## Discussion and conclusion

This study presented ROCIP, which fuses a DenseNet based neural statistical motion model and a RBPF for long-term inertial tracking with stable errors. The proposed system was trained and evaluated with a newly collected dataset. It outperformed the comparative methods in both ATE and RTE, and generated estimated trajectories that are much closer to the ground truth. This success is primarily attributed to the design of a system that effectively integrates a probabilistic neural network with a particle filter. This integration is achieved by leveraging the neural network’s predictions and uncertainties directly in the particle filter, where they are used as parameters of a Gaussian distribution for particle sampling. By doing so, the particle filter effectively accounts for variability and noise in the motion data, enabling sampling accuracy. This seamless connection between the two components allows for more robust long-term tracking and error correction, which is particularly challenging in head-mounted inertial tracking scenarios.

The result also shows the robustness of the proposed system in short-term and long-term tracking. ATE is the RMSE of the total trajectory, which is around 7-12 minutes in this study. RTE could be considered as the ATE in one minute. The ATEs of the PDR and HINNet were around a factor of two larger than their RTEs, which suggests that their errors are gradually accumulating as time increases. However, the proposed ROCIP has similar RTE and ATE, indicating that ROCIP error remains relatively stable even during long-term tracking. Figure 4 also provides further evidence of this. The trajectories generated by HINNet and PDR are gradually getting further and further away from the ground truth because of the error accumulations and the lack of calibration. However, the trajectories estimated by ROCIP remain close to the ground truth as time goes on. ROCIP demonstrates strong performance across all four scenarios, providing clear evidence of its multi-environment tracking stability.

An interesting observation regarding the distance error is that the error after the neural network is only 0.23%, whereas it increases to 2.81% after the application of the particle filter. This discrepancy likely arises from the sampling process in the particle filter. During sampling, the distance between a particle’s current state and its previous state does not strictly match the predicted distance from the neural network, $\Delta l$. Instead, it follows a Gaussian distribution with a mean of $\Delta l$ and a standard deviation of $\sigma _{\Delta l}$. While the deviations help the particles adhere to the prior trajectory and improve robustness, they also introduce a divergence from the neural network’s direct predictions, leading to the observed difference in distance error.

The probabilistic neural network also proves its ability to give reasonable uncertainty estimations. Figure 5 shows the estimation result on walking and running at various speeds. 4 walking periods (0-110*s*, 190-220*s*, 290-320*s*, 370-420*s*) show similar speeds around 3*m*/*s*, while 3 running periods (110-190*s*, 220-290*s*, 320-370*s*) show increasing speeds (4*m*/*s*, 5*m*/*s*, 6*m*/*s*). With the speed increasing, the estimated uncertainty (light red region in the figure) shows a trend to increase as well, which infers that the estimation of neural network has larger uncertainty for higher speed. Figure 6 shows the estimation result for walking with a constant speed around 2.5-3*m*/*s*. The uncertainty also remains in a relatively constant range, and clearly much smaller than that of the running periods in Fig. 5.

The variation in uncertainty ($\sigma$) arises from several possible contributing factors:

- **Complex and Dynamic Movements:** Running introduces more intricate and unpredictable motion patterns compared to walking. The higher acceleration and rapid changes in direction generate increased noise in the IMU signals, which inherently leads to larger uncertainty estimates by the neural network. This reflects the network’s sensitivity to the variability and complexity of the input data.
- **Impact of Coordinate Transformation:** The coordinate transformation process relies on updating roll and pitch angles based on the product of angular velocity and time, as well as the assumption that gravity dominates the acceleration signal. During periods of intense activity, such as running, the acceleration signal may deviate from this assumption due to the dominance of other dynamic forces. This deviation introduces additional noise into the transformation process, compounding the uncertainty.
- **Motion Artifacts:** The physical movement of the head-mounted IMU, including minor shifts or displacements of the strap during high-intensity motion, may create motion artifacts. These artifacts add further irregularities to the sensor data, amplifying uncertainty during activities involving significant physical exertion.

This study also proposed a new larger dataset including more subjects, more scenarios, more motions, and speeds. Comparing HINNet results on this new dataset and on the dataset proposed in [18], it shows worse performance in a larger dataset, which seems counter-intuitive. But it is because the new dataset includes more complex motions such as walking and running at different speeds, and the scenarios are much larger (150*80*m*) comparing to previous small maps (15*20*m*). Although accuracy decreased, robustness improved, allowing the system to work with more complex motions and environments.

The proposed method has broad applications in fields where low-cost, unobtrusive, and infrastructure-free tracking is essential. Examples include: (i) monitoring the movements and locations of patients or elderly individuals living alone, using IMUs embedded in everyday items like glasses, hearing aids, or dental appliances; (ii) tracking movements and trajectories during sports activities with IMUs integrated into devices such as earphones, helmets, or mouthguards; and (iii) providing reliable, self-contained navigation for rescuers in scenarios where GPS or other positioning systems are unavailable, such as disaster zones or remote areas.

These kind of approaches can also be generalized to other research areas, such as the tracking of legged robots. The models are also strong candidates for monitoring, e.g., professionals or robots during search and rescue missions, in mining or agriculture [32]. As these environments could require robots that can cope with obstacles, rough outdoor terrain, and steps, which is difficult for wheeled robots. Using IMUs with algorithms similar to human tracking could serve as a component in the fusion of its multi-sensor localization.

As for the future direction, using larger models and dataset is an inevitable trend in deep learning. Increasing the scale of the model parameters will allow more complex motion interpretations in higher dimensions. Recently ChatGPT has become highly influential and could do complex text task, which is mostly attributed to the large GPT-3 model of 175 billion parameters and a training dataset with 499 Billion tokens [6]. However the model in this study only used 669,592 parameters. Reflecting again on the deep learning for human motion, a variety of tasks that are currently not considered could be fulfilled if a similar size of model and dataset would be adopted.

## Data Availability

Code and dataset can be found in https://github.com/Oxford-NIL/ROCIP.

## References

[1] Beuchert J, Camurri M, Fallon M (2023) Factor graph fusion of raw gnss sensing with imu and lidar for precise robot localization without a base station. In: 2023 IEEE international conference on robotics and automation (ICRA). IEEE, pp 8415–8421

[2] Romme J, Heuvel J, Dolmans G, Selimis G, Philips K, De Groot H (2014) Measurement and analysis of UWB radio channel for indoor localization in a hospital environment. In: 2014 IEEE international conference on ultra-wideband (ICUWB). IEEE, pp 274–279

[3] Beuchert J, Rogers A (2021) Snappergps: algorithms for energy-efficient low-cost location estimation using gnss signal snapshots. In: Proceedings of the 19th ACM conference on embedded networked sensor systems, pp 165–177

[4] Ferreira AFGG, Fernandes DMA, Catarino AP, Monteiro JL (2017) Localization and positioning systems for emergency responders: a survey. IEEE Commun Surv Tutor 19(4):2836–2870

[5] Hou X, Bergmann J (2020) A pedestrian dead reckoning method for head-mounted sensors. Sensors 20(21):6349 33171710 10.3390/s20216349 PMC7664376

[6] Brown T, Mann B, Ryder N, Subbiah M, Kaplan JD, Dhariwal P, Neelakantan A, Shyam P, Sastry G, Askell A et al (2020) Language models are few-shot learners. Adv Neural Inf Process Syst 33:1877–1901

[7] Noh H, Hong S, Han B (2015) Learning deconvolution network for semantic segmentation. In: Proceedings of the IEEE international conference on computer vision, pp 1520–1528

[8] Hou X, You J, Hu P (2019) Predicting drug-drug interactions using deep neural network. In: Proceedings of the 2019 11th international conference on machine learning and computing, pp 168–172

[9] Yang W, Sparrow SN, Wallom DC (2023) Optimising multi-factor assistance in a deep learning-based electricity forecasting model with climate resilience: an australian case study. In: 2023 IEEE PES innovative smart grid technologies europe (ISGT EUROPE). IEEE, pp 1–5

[10] Stephen O, Sain M, Maduh UJ, Jeong D-U, et al (2019) An efficient deep learning approach to pneumonia classification in healthcare. J Healthcare Eng 2019 10.1155/2019/4180949 PMC6458916 31049186

[11] Chen C, Lu X, Markham A, Trigoni N (2018) Ionet: learning to cure the curse of drift in inertial odometry. In: Proceedings of the AAAI conference on artificial intelligence, vol 32

[12] Yan H, Shan Q, Furukawa Y (2018) Ridi: robust imu double integration. In: Proceedings of the european conference on computer vision (ECCV), pp 621–636

[13] Herath S, Yan H, Furukawa Y (2020) Ronin: robust neural inertial navigation in the wild: benchmark, evaluations, & new methods. In: 2020 IEEE international conference on robotics and automation (ICRA). IEEE, pp 3146–3152

[14] Liu W, Caruso D, Ilg E, Dong J, Mourikis AI, Daniilidis K, Kumar V, Engel J (2020) Tlio: tight learned inertial odometry. IEEE Robot Autom Lett 5(4):5653–5660

[15] Song Y, Xia S, Yang J, Pei L (2024) A learning-based multi-node fusion positioning method using wearable inertial sensors. In: ICASSP 2024-2024 IEEE international conference on acoustics, speech and signal processing (ICASSP). IEEE, pp 1976–1980

[16] Bergmann JH, Chandaria V, McGregor A (2012) Wearable and implantable sensors: the patient’s perspective. Sensors 12(12):16695–16709 23443394 10.3390/s121216695 PMC3571806

[17] Hou X, Bergmann J (2020) Pedestrian dead reckoning with wearable sensors: a systematic review. IEEE Sens J 21(1):143–152

[18] Hou X, Bergmann JH (2023) Hinnet: inertial navigation with head-mounted sensors using a neural network. Eng Appl Artif Intell 123:106066

[19] Hou X, Bergmann J (2023) Hinnet + headslam: robust inertial navigation with machine learning for long-term stable tracking. IEEE Sens Lett 7(8):1–4. 10.1109/LSENS.2023.329455337529707

[20] Russell RL, Reale C (2022) Multivariate uncertainty in deep learning. IEEE Trans Neural Netw Learn Syst 33(12):7937–7943. 10.1109/TNNLS.2021.3086757 34138723 10.1109/TNNLS.2021.3086757

[21] Kendall A, Gal Y (2017) What uncertainties do we need in bayesian deep learning for computer vision? Adv Neural Inf Process Syst 30

[22] Bergmann JH, Langdon PM, Mayagoitia RE, Howard N (2014) Exploring the use of sensors to measure behavioral interactions: an experimental evaluation of using hand trajectories. PLoS One 9(2):88080 10.1371/journal.pone.0088080 PMC3917885 24516583

[23] Yu X, Liu B, Lan X, Xiao Z, Lin S, Yan B, Zhou L (2019) Azupt: adaptive zero velocity update based on neural networks for pedestrian tracking. In: 2019 IEEE global communications conference (GLOBECOM), pp 1–6. 10.1109/GLOBECOM38437.2019.9014070

[24] Yun X, Bachmann ER, Moore H, Calusdian J (2007) Self-contained position tracking of human movement using small inertial/magnetic sensor modules. In: Proceedings 2007 IEEE international conference on robotics and automation, pp 2526–2533. 10.1109/ROBOT.2007.363845

[25] Angermann M, Robertson P (2012) Footslam: pedestrian simultaneous localization and mapping without exteroceptive sensors–hitchhiking on human perception and cognition. Proc IEEE 100(Special Centennial Issue):1840–1848

[26] Hou X, Bergmann J (2022) Headslam: pedestrian slam with head-mounted sensors. Sensors 22(4):1593 35214500 10.3390/s22041593 PMC8875564

[27] Huang G, Liu Z, Van Der Maaten L, Weinberger KQ (2017) Densely connected convolutional networks. In: Proceedings of the IEEE conference on computer vision and pattern recognition, pp 4700–4708

[28] He K, Zhang X, Ren S, Sun J (2016) Deep residual learning for image recognition. In: Proceedings of the IEEE conference on computer vision and pattern recognition, pp 770–778

[29] Montemerlo M, Thrun S, Koller D, Wegbreit B, et al (2002) Fastslam: a factored solution to the simultaneous localization and mapping problem. AAAI/IAAI 593598

[30] Richards A (2015) University of Oxford advanced research. Computing. 10.5281/zenodo.22558

[31] Zhang Z, Scaramuzza D (2018) A tutorial on quantitative trajectory evaluation for visual (-inertial) odometry. In: 2018 IEEE/RSJ international conference on intelligent robots and systems (IROS). IEEE, pp 7244–7251

[32] Bellicoso CD, Bjelonic M, Wellhausen L, Holtmann K, Günther F, Tranzatto M, Fankhauser P, Hutter M (2018) Advances in real-world applications for legged robots. J Field Robot 35(8):1311–1326

